# Hem coagulase induced cerebral venous sinus thrombosis in patients with uterine fibroids surgery

**DOI:** 10.1097/MD.0000000000032948

**Published:** 2023-02-17

**Authors:** Bei Sun, Tingting Liu, Bingchao Xu, Guanghui Zhang, Kang Xie

**Affiliations:** a Department of Neurosurgery, Lianyungang Hospital Affiliated to Xuzhou Medical University, Lianyungang, China.

**Keywords:** cerebral venous sinus thrombosis, CT, hem coagulase, MRI, uterine fibroids

## Abstract

Cerebral venous sinus thrombosis (CVST) is a rare, high-risk, and easily misdiagnosed disease. Currently, there are case reports of hem coagulase-induced thrombotic events, but no reports of CVST being associated with hem coagulase.

**Case summary:**

A 35-years-old woman presented to the outpatient clinic with a severe headache and sudden memory loss with intravenous hem coagulase for postoperative bleeding after uterine fibroids surgery. Abnormal neurological signs included slowed reactions, poor memory, and decreased numeracy. Magnetic resonance imaging and computed tomography scan showed multiple cerebral infarcts, and the infarct area was non-arterial. Brain magnetic resonance venography showed obstruction of the left sigmoid sinus. High-resolution magnetic resonance imaging of the left sigmoid sinus showed abnormally high signal. The patient was treated with a subcutaneous Low-Molecular-Weight Heparin Sodium injection 0.4 ml, twice a day (7 days), and oral Warfarin Sodium 3 mg, once a day, while monitoring the international normalized ratio, adjust the warfarin sodium dosage according to the international normalized ratio level. One month later, the patient had no neurological symptoms and her cognitive function returned to normal.

**Conclusion:**

hem coagulase may be a contributing factor to CVST in patients undergoing uterine fibroids surgery and should be administered intravenously with caution.

## 1. Introduction

Cerebral venous sinus thrombosis (CVST), a special type of venous thrombosis, is a rare but potentially life-threatening disease that occurs when cerebral venous blood flow is blocked by a thrombus and causes severe cerebral edema, cerebral infarction, and hemorrhagic transformation.^[1]^ Symptoms of CVST are often variable and typically present with headache, paralysis, seizures, papilledema, psych behavioral abnormalities, and disturbance of consciousness.^[2]^ The main risk factors for CVST are: oral contraceptives, pregnancy, puerperium, trauma, surgery, cancer, blood diseases, etc.^[3]^

The etiology of CVST is complex and diverse and includes multiple drugs such as oral contraceptives, L-asparaginase, ecstasy,^[1]^ and sildenafil.^[4]^ The patient in this case did not take any related medications and had no other possible risk factors. According to the current research progress, there is no report related to CVST and hem coagulase. However, some other types of thrombotic events are induced by hem coagulase. Abdominal surgery usually causes hypercoagulable state, and use of hem coagulase may aggravate hypercoagulability, even increase the incidence of deep venous thrombosis in lower limbs.^[5]^ A patient with upper gastrointestinal bleeding developed a massive cerebral infarction after receiving intravenous hem coagulase.^[6]^ These reports make clinicians begin to pay attention to the use of hem coagulase. Here, we reported a rare case of a 35-years-old woman who presented with CVST following hem coagulase therapy after uterine fibroids, which shows for the first time that hem coagulase can cause CVST.

## 2. Case presentation

A 35-years-old woman complained of severe headache and sudden memory loss. Twelve days ago, she underwent uterine fibroids surgery under general anesthesia. Subsequently, due to postoperative bleeding, the patient received intravenous hem coagulase (2 IU/day) for 7 consecutive days. Three days ago, she developed severe headaches and memory loss. The patient reported no nausea, vomiting, photophobia, or blurred vision.

The patient had a clear previous medical history with a 2-year history of uterine fibroids, without taking any oral medication. Physical examination was unremarkable except for an incision in the lower abdomen. Abnormal neurological signs include the following: slow reactions, poor memory, and decreased ability to perform calculations. The patient limbs had normal muscle strength and motor coordination. No nuchal rigidity or other signs of meningitis were identified.

Routine blood tests were normal, and APTT and international normalized ratio (INR) were normal. Serum D-dimer level was 908 ng/mL. Hepatic enzymogram was slightly increased. The levels of serum tumor markers, such as cancer antigen-125 (CA-125), antigen-CA153, antigen-CA199, α-fetoprotein, carcinoembryonic antigen, ferritin, and neuron-specific enolase were within normal ranges.

**Figure 1.**
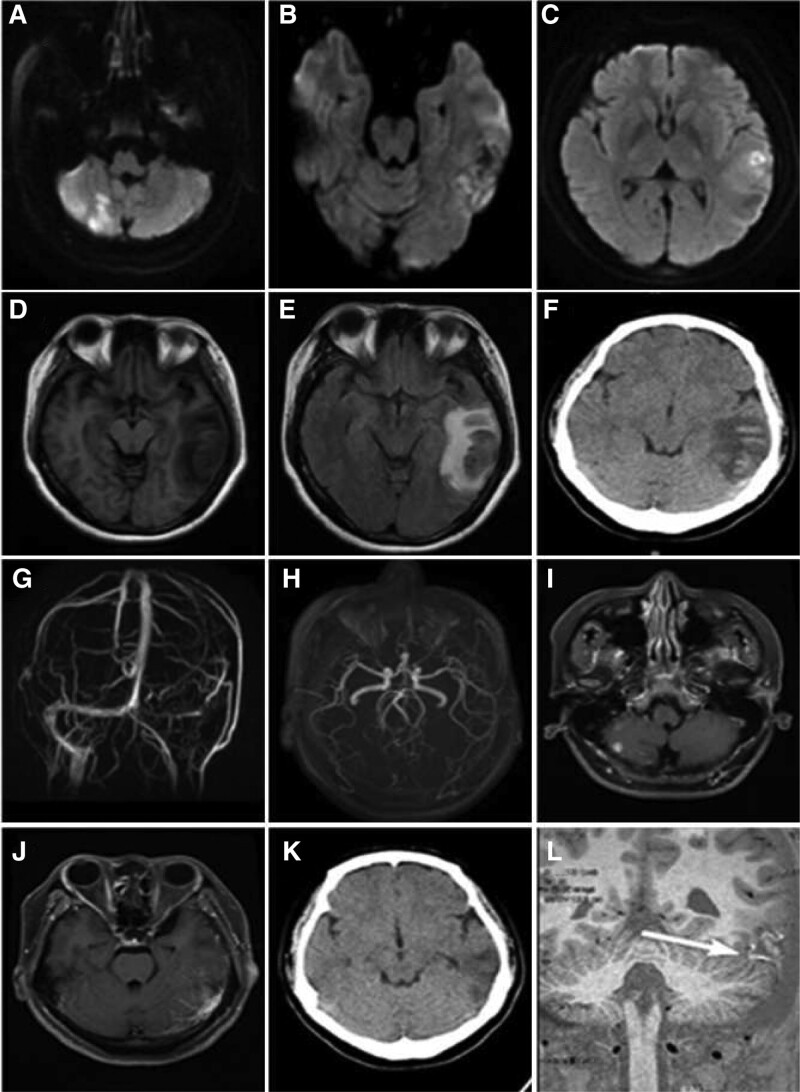
Brain imaging findings before and after treatment. (A, B and C) Diffusion-weighted imaging showed multiple infarcts. (D and E) T1-weighted images (T1WI) and Fluid-attenuated inversion-recovery (FLAIR) images showed heterogeneous signal and distinct edema in the left temporal lobe. (F) CT scan showed hemorrhagic infarction in the left temporal lobe. (G) Magnetic resonance venography (MAV) demonstrated a vague left sigmoid sinus. (H) Magnetic resonance angiography (MRA) showed no arterial vascular stenosis or occlusion. (I and J) Cranial contrast-enhanced MRI revealed cord-like enhanced signals in the left temporal lobe and a strong signal in the left sigmoid sinus. (K) Follow-up CT scan showed the disappearance of high density in the left temporal lobe after a month. (L) High resolution MRI with T1WI 3D-VISTA displayed chronic thrombosis in the left sigmoid sinus, white arrow represented sigmoid sinus. MRI = magnetic resonance imaging.

Cranial magnetic resonance imaging (MRI) showed acute multiple infarcts in the right cerebellar hemisphere and left temporal lobe (Fig. 1A–1C). *T*1-weighted images and *T*2-weighted images/fluid-attenuated inversion-recovery showed marked edema and heterogeneous signal in the left temporal lobe (Fig. 1D and 1E) by subsequent computed tomography (CT). Hemorrhagic infarction was further identified (Fig. 1F). Magnetic resonance venography of the brain showed a vague left sigmoid sinus (Fig. 1G). Magnetic resonance angiography (MRA) showed no arterial vascular stenosis or occlusion (Fig. 1H). Cranial contrast-enhanced MRI showed a cord-like enhanced signals in the left temporal lobe and a strong signal in the left sigmoid sinus (Fig. 1I and 1J), as the results of contrast agent retention. No tumor-like enhanced signs were found. Contrast-enhanced CT of the chest and abdomen showed no abnormality. The final diagnosis in this case was CVST.

The patient received a subcutaneous injection of Low-Molecular-Weight Heparin Sodium 0.4 mL, twice a day (7 days), and oral Warfarin Sodium 3 mg, once a day, while monitoring the INR (international normalized ratio), adjust the warfarin sodium dosage according to the INR level. Dehydrating agents were used to decrease the intracranial pressure.

One week later, the patient headache was relieved and her cognitive function improved significantly. One month later, a follow-up MRI showed a decrease in the area of mixed signal in the left temporal lobe. A cranial CT scan showed that the high density in the left temporal lobe had disappeared (Fig. 1K). High-resolution MRI with T1-weighted 3D volumetric isotropic turbo spin echo acquisition clearly displayed abnormal high signal in the left sigmoid sinus (Fig. 1L). The patient was asymptomatic and had normal cognitive assessments.

## 3. Discussion

In this case, the patient presented with severe headache and mild cognitive impairment. Neuroimaging showed infarcts in multiple non-arterial vascular areas. Cranial magnetic resonance venography showed obstruction of the left sigmoid sinus. High-resolution magnetic resonance imaging clearly demonstrated left sigmoid sinus thrombosis. In addition, since no abnormality of cerebral artery system was found in the cranial MRA, the right cerebellar infarction may be of venous origin. As a result, the patient was diagnosed with CVST.

Hem coagulase is commonly used to prevent postoperative bleeding or treat systemic bleeding, especially in China.^[7–10]^ As an activator of coagulation factor X, hem coagulase plays an extremely important role in hemostasis. Although its concentration in serum is low, it has a strong coagulation effect through a cascade reaction in the body. It can hydrolyze fibrinogen, catalyze the production of thrombin in damaged blood vessels, promote the production of factor Xa, and effectively activate prothrombin.^[11]^ We speculate that the use of hem coagulase may promote platelet aggregation or activate the coagulation system, resulting in a hypercoagulable state of cerebral vascular sinuses.

## 4. Conclusion

In this case, a woman who underwent uterine fibroids surgery developed CVST after intravenous injection with hem coagulase. Therefore, hem coagulase may be a contributing factor for CVST, and should be cautiously used via intravenous injection.

## Author contributions

**Conceptualization:** Guanghui Zhang, Kang Xie.

**Formal analysis:** Bei Sun, Tingting Liu.

**Resources:** Guanghui Zhang.

**Supervision:** Kang Xie.

**Writing – original draft:** Bei Sun, Tingting Liu, Bingchao Xu.
